# Balanced crystalloids for septic shock resuscitation

**DOI:** 10.5935/0103-507X.20160079

**Published:** 2016

**Authors:** Thiago Domingos Corrêa, Alexandre Biasi Cavalcanti, Murillo Santucci Cesar de Assunção

**Affiliations:** 1 Intensive Care Unit, Hospital Israelita Albert Einstein - São Paulo (SP), Brazil.; 2 Research Institute, Hospital do Coração - São Paulo (SP), Brazil.

**Keywords:** Fluid therapy/methods, Isotonic solutions/administration & dosage, Rehydration solutions/administration & dosage, Shock, septic, Resuscitation/methods, Critical care/methods, Critical care/trends, Hidratação/métodos, Soluções isotônicas/administração & dosagem, Soluções para reidratação/administração & dosagem, Choque séptico, Ressuscitação/métodos, Cuidados críticos/métodos, Cuidados críticos/tendências

## Abstract

Timely fluid administration is crucial to maintain tissue perfusion in septic shock patients. However, the question concerning which fluid should be used for septic shock resuscitation remains a matter of debate. A growing body of evidence suggests that the type, amount and timing of fluid administration during the course of sepsis may affect patient outcomes. Crystalloids have been recommended as the first-line fluids for septic shock resuscitation. Nevertheless, given the inconclusive nature of the available literature, no definitive recommendations about the most appropriate crystalloid solution can be made. Resuscitation of septic and non-septic critically ill patients with unbalanced crystalloids, mainly 0.9% saline, has been associated with a higher incidence of acid-base balance and electrolyte disorders and might be associated with a higher incidence of acute kidney injury. This can result in greater demand for renal replacement therapy and increased mortality. Balanced crystalloids have been proposed as an alternative to unbalanced solutions in order to mitigate their detrimental effects. Nevertheless, the safety and effectiveness of balanced crystalloids for septic shock resuscitation need to be further addressed in a well-designed, multicenter, pragmatic, randomized controlled trial.

## Abstract (pt)

A administração de fluidos em tempo adequado é crucial para a manutenção da perfusão tissular nos pacientes com choque séptico. Entretanto, a questão da escolha do fluido a ser utilizado para ressuscitação no choque séptico ainda é um assunto em debate. É crescente o corpo de evidência que sugere que o tipo, a quantidade e o momento da administração de fluidos durante a evolução da sepse podem afetar os desfechos do paciente. Os cristaloides têm sido recomendados como fluidos a serem administrados em primeira linha na ressuscitação do choque. No entanto, à luz da natureza inconclusiva da literatura disponível, não se podem fazer recomendações definitivas quanto à solução cristaloide mais apropriada. A ressuscitação de pacientes críticos sépticos e não sépticos com cristaloides não balanceados, principalmente a solução salina a 0,9%, tem sido associada a uma maior incidência de desordens do equilíbrio ácido-base e a distúrbios eletrolíticos, além de poder se associar à maior incidência de lesão renal aguda, à maior necessidade de terapia de substituição renal e à mortalidade. Foi proposto o uso de soluções cristaloides balanceadas como uma alternativa às soluções de cristaloides não balanceados, para mitigar seus efeitos deletérios. Entretanto, a segurança e a eficácia dos cristaloides balanceados para ressuscitação do choque séptico necessitam ser mais bem exploradas em estudos clínicos bem delineados, randomizados e controlados, multicêntricos e pragmáticos.

## INTRODUCTION

Septic shock is characterized by intense systemic vasodilation with varying degrees of hypovolemia.^(1)^ Timely fluid administration is crucial to improve cardiac output, restore oxygen delivery and reverse tissue hypoxia.^(1)^ As a result, cellular and mitochondrial dysfunction as well as progression to multiple organ dysfunction syndrome secondary to systemic inflammation and tissue hypoperfusion are mitigated.^(1)^ Therefore, fluid administration is recommended as a first-line intervention to resuscitate septic shock patients.^(2)^

A growing body of evidence suggests that the type, amount and timing of fluid administration during the course of sepsis may affect patient outcomes.^(3)^ While early fluid administration has been associated with decreased in-hospital mortality,^(4)^ delayed resuscitation has been associated with a pronounced release of inflammatory mediators and decreased skeletal muscle adenosine triphosphate content and mitochondrial dysfunction.^(5)^ Furthermore, liberal fluid administration to septic shock patients yields a net positive fluid balance, which may contribute to organ failure and poor outcomes.^(6)^

Many types of fluids are available for clinicians at the bedside.^(3)^ Nevertheless, since the type and amount of administered fluids affect patient-centered outcomes,^(7,8)^ such drugs should be prescribed with caution.^(3)^ Moreover, it is important to emphasize that fluid administration should be indicated only for those who have impaired tissue perfusion and are deemed fluid responsive (i.e., patients with a high likelihood of improving cardiac output after fluid administration).^(9)^ Whenever fluids are judged necessary, clear endpoints of efficacy and safety must be defined in advance in order to maximize the efficacy of the fluids administered and minimize their potential detrimental effects.^(9)^

The question concerning which fluid should be used during septic shock resuscitation remains a matter of debate.^(10)^ The current Surviving Sepsis Campaign Guidelines recommend crystalloids as first-line fluids for septic shock resuscitation.^(2)^ Nevertheless, no consensus has been reached regarding which crystalloid, i.e., unbalanced or balanced, is the most appropriate in this context.^(11)^

This narrative review briefly discusses the main physicochemical properties of unbalanced and balanced crystalloids as well as their main advantages and drawbacks. It also presents evidence supporting balanced crystalloids as the fluids of choice for septic shock resuscitation.

This narrative literature review includes articles published in the MEDLINE/PubMed database until March 2016 that addressed crystalloid fluid resuscitation in critically ill patients. We used the search term "balanced solution" and search filters for systematic reviews (systematic [sb]) and randomized trials (((clinical[Title/Abstract] AND trial[Title/Abstract]) OR clinical trials as topic[MeSH Terms] OR clinical trial[Publication Type] OR random*[Title/Abstract] OR random allocation[MeSH Terms] OR therapeutic use[MeSH Subheading])).^(12)^ The search retrieved 433 references. After title and abstract screening, we selected the full-text versions of 95 relevant citations for a thorough analysis. We also searched reference lists of the retrieved manuscripts to identify other relevant studies.

## CRYSTALLOIDS

Solutions containing water and freely permeable ions, mainly sodium and chloride, are classified as crystalloids (Table 1).^(3)^ Some of these solutions have other ions, such as potassium, calcium or magnesium, and may have buffers, most commonly bicarbonate, lactate, acetate or gluconate, to maintain electroneutrality (balance between positive and negative ions).^(3)^ Crystalloid solutions may be hypotonic, isotonic or hypertonic in relation to human plasma.^(3)^ A crystalloid solution is considered balanced when it has a strong ion difference (SID) close to 24mEq/L,^(13)^ which can be achieved by replacing varying amounts of chloride from 0.9% saline with bicarbonate, lactate or acetate (Table 1).^(3)^

**Table 1:** Composition of the available unbalanced and balanced crystalloids

Composition/properties	Solutions						
	Human plasma	0.9% saline	Ringer’s solution	Hartmann’s solution	Ringer’s lactate	Ringer’s acetate	Plasma-Lyte
pH	7.35 - 7.45	5.5	6.0	6.5	6.5	6.7	7.4
Osmolality (mOsm/L)	291	308	310	279	273	270	294
Sodium (mmol/L)	135 - 145	154	147	131	130	131	140
Potassium (mmol/L)	4.5 - 5.5		4	5	4	4	5
Calcium (mmol/L)	2.2 - 2.6		2.2	2	1.5	2	
Magnesium (mmol/L)	0.8 - 1.0					1	1.5
Chloride (mmol/L)	94 - 111	154	156	111	109	110	98
Bicarbonate (mmol/L)	23 - 27						
Lactate (mmol/L)	1.0 - 2.0			29	28		
Acetate (mmol/L)						30	27
Gluconate (mmol/L)							23

### Unbalanced crystalloids

Sodium chloride (0.9% saline) is the most available and frequently used crystalloid worldwide.^(3)^ Sodium chloride is an isotonic solution (osmolality closer to that of human plasma) that contains equal concentrations of sodium and chloride (154mmol/L, each) and therefore has an SID equal to zero (Table 1). Experimental^(14-22)^ (Table 2) and clinical^(23-37)^ studies (Table 3) have suggested that resuscitation with 0.9% saline has detrimental effects on the kidneys, acid-base balance, and electrolyte homeostasis and may affect tissue perfusion,^(38)^ inflammatory response,^(14)^ and coagulation (dilutional coagulopathy and/or profound hyperchloremic metabolic acidosis).^(27,39)^

**Table 2:** Summary of experimental studies comparing balanced with unbalanced crystalloids

Author, year	Experimental model	Comparisons*	Main study findings
Zhou et al.^(14)^	Abdominal sepsis in rats	0.9% saline Plasma-Lyte	Higher serum chloride and lower pH with 0.9% saline than with Plasma-Lyte. Higher incidence of AKI and lower survival with 0.9% saline compared to Plasma-Lyte.
Healey et al.^(15)^	Moderate hemorrhage and massive hemorrhage (35% and 218% of TBV removed, respectively) in rats	0.9% saline Ringer’s lactate	No acid-base difference in moderate hemorrhage. In massive hemorrhage, less acidosis and improved survival with Ringer’s lactate compared to 0.9% saline.
Watters et al.^(16)^	Uncontrolled hemorrhagic shock in pigs	0.9% saline Ringer’s Lactate	Lung inflammation was similar to 0.9% saline and Ringer’s lactate.
Todd et al.^(17)^	Uncontrolled hemorrhagic shock in pigs	0.9% saline Ringer’s lactate	Less Ringer’s lactate than 0.9% saline was necessary to restore baseline MAP. Higher urinary output with 0.9% saline than Ringer’s lactate. Higher incidence of hyperchloremic acidosis and lower fibrinogen levels with 0.9% saline than with Ringer’s lactate.
Noritomi et al.^(18)^	Hemorrhagic shock in pigs (40% of TBV removed)	0.9% saline Ringer’s lactate Plasma-Lyte	Increased base excess and lower chloride levels with Ringer’s lactate and Plasma-Lyte compared to 0.9% saline. Unmeasured anions did not differ between the groups.
Rohrig e Rönn.^(19)^	Severe hemorrhagic shock in rats	Ringer’s lactate Ringer’s solution	Higher survival and lower incidence of AKI with Ringer’s solution than with Ringer’s lactate.
Aksu et al.^(20)^	Hemorrhagic shock in rats	0.9% saline Plasma-Lyte	Renal blood flow and kidney oxygen consumption improved with Plasma-Lyte compared to 0.9% saline. Resuscitation with Plasma-Lyte prevented hyperchloremia, restored acid-base balance and preserved SID. No difference in systemic inflammation nor in oxidative stress.
Martini et al.^(21)^	Severe hemorrhagic shock (60% of TBV removed) in pigs	0.9% saline Ringer’s lactate	Lower volume of Ringer’s lactate than 0.9% saline necessary to restore baseline MAP. Base excess restored with Ringer’s lactate but not with 0.9% saline. Similar effects on coagulation. Serum potassium increased with 0.9% saline while unaffected with Ringer’s Lactate.
Rohrig et al.^(22)^	Severe hemorrhagic shock in rats	0.9% saline Ringer’s lactate Ringer’s acetate Ringer’s solution	Lower survival with Ringer’s lactate than with all other solutions. Pronounced metabolic acidosis with 0.9% saline and Ringer’s solutions than with Ringer’s lactate and Ringer’s acetate.

AKI - acute kidney injury; TBV - total blood volume; MAP - mean arterial blood pressure; SID - strong ion difference.

* comparisons other than 0.9% saline versus balanced solutions were not considered.

**Table 3:** Summary of randomized controlled trials comparing balanced with unbalanced crystalloids in clinical-surgical critically ill patients

Author, year	N	Patients	Comparisons*	Main study findings
McFarlane e Lee^(23)^	30	Open abdominal surgery	0.9% saline Plasma-Lyte	Acidosis and hyperchloremia with 0.9% saline.
Waters et al.^(24)^	66	Aortic reconstructive surgery	0.9% saline Ringer’s lactate	Acidosis, hyperchloremia and increased need of platelets and blood products transfusion with 0.9% saline.
Takil et al.^(25)^	30	Spinal surgery	0.9% saline Ringer’s lactate	Acidosis and hyperchloremia with 0.9% saline. Respiratory acidosis and mild hyponatremia with Ringer’s lactate.
Young et al.^(26)^	46	Trauma	0.9% saline Plasma-Lyte	Acidosis and hyperchloremia with 0.9% saline.
Smith et al.^(27)^	18	Trauma	0.9% saline Plasma-Lyte	Acidosis with 0.9% saline. Faster clot formation with Plasma-Lyte.
O’Malley et al.^(28)^	51	Kidney transplantation	0.9% saline Ringer’s lactate	Acidosis and hyperkalemia with 0.9% saline.
Khajavi et al.^(29)^	52	Kidney transplantation	0.9% saline Ringer’s lactate	More acidosis and hyperkalemia with 0.9% saline than with Ringer’s lactate.
Hadimioglu et al.^(30)^	90	Kidney transplantation	0.9% saline Ringer’s lactate Plasma-Lyte	Hyperchloremic acidosis with 0.9% saline. Increased lactate levels with Ringer’s lactate. Potassium levels unchanged in all groups.
Modi et al.^(31)^	74	Kidney transplantation	0.9% saline Ringer’s lactate	Acidosis, hyperchloremia and hyperkalemia with 0.9% saline.
Kim et al.^(32)^	60	Kidney transplantation	0.9% saline Plasma-Lyte	Acidosis and hyperchloremia with 0.9% saline.
Mahler et al.^(33)^	45	Diabetic ketoacidosis	0.9% saline Plasma-Lyte	Hyperchloremic metabolic acidosis with 0.9% saline.
Van Zyl et al.^(34)^	54	Diabetic ketoacidosis	0.9% saline Ringer’s lactate	Longer time to reach a blood glucose level of 14 mmol/L with Ringer’s lactate.
Hasman et al.^(35)^	90	Moderate or severe dehydration	0.9% saline Ringer’s lactate Plasma-Lyte	Pronounced acidosis with 0.9% saline.
Cieza et al.^(36)^	40	Severe dehydration	0.9% saline Ringer’s lactate	Acidosis and hyperchloremia with 0.9% saline.
Wu et al.^(37)^	40	Acute pancreatitis	0.9% saline Ringer’s lactate	Acidosis and hyperchloremia with 0.9% saline. Lower incidence of SIRS and lower CRP levels 24 hours after randomization in Ringer’s lactate compared to 0.9% saline.

SIRS - systemic inflammatory response syndrome; CRP - C-reactive protein.

* comparisons other than 0.9% saline versus balanced solutions were not considered.

Hyperchloremia adversely affects kidney function.^(40)^ Intrarenal (kidney artery) infusion of chloride-containing solutions, such as 0.9% saline or ammonium chloride (NH_4_Cl), leads to a reduced renal artery blood flow and glomerular filtration rate in kidneys isolated from healthy dogs.^(41)^ Intravascular volume expansion with solutions containing supraphysiological chloride concentrations, such as 0.9% saline, leads to increased chloride delivery to the macula densa cells localized to the distal nephrons.^(40)^ As a result, a number of signaling mediators, such as adenosine, are released from macula densa cells into the kidney circulation (tubuloglomerular feedback).^(42)^ Adenosine has a strong constrictive effect on the renal afferent arteriole, which compromises the renal blood flow, glomerular filtration rate and, ultimately, kidney function.^(40)^

The impact of intravascular volume expansion with unbalanced solutions (0.9% saline) containing supraphysiological amounts of chloride on acid-base balance and electrolyte homeostasis are better explained by the physicochemical Stewart approach.^(43)^ Accordingly, strong cations (Na^+^, K^+^, Mg^2+^ and Ca^2+^) predominate in relation to strong anions (Cl^-^) in the body, producing a net positive plasma charge of approximately 40mmol/L, which is also known as the SID.^(43)^ This positive plasma charge must be counterbalanced with an equal negative charge to sustain the electrical neutrality (Law of electro-neutrality). The balancing anionic charge is derived from nonvolatile weak acids, mainly albumin and phosphate.^(43)^ Infusion with large amounts of 0.9% saline produces hyperchloremic metabolic acidosis in healthy volunteers and in different populations of critically ill patients (Table 3).

Hyperchloremic metabolic acidosis occurs because 0.9% saline contains strong cations and strong anions in the same quantity (SID equal to zero). When the plasma chloride concentration increases after 0.9% saline infusion, the net positive charge of plasma (SID) is reduced. Conversely, compensatory mechanisms designed to maintain the plasma electro-neutrality are activated, thus increasing the plasma positive charge (H^+^) while decreasing the arterial pH.^(43)^

### Balanced crystalloids

Balanced crystalloids have been proposed as an alternative to unbalanced solutions in order to mitigate their detrimental effects.^(3)^ The most commonly available balanced crystalloids are presented in table 1. Ringer's lactate is produced by adding sodium lactate as a buffer to Ringer's solution to reduce its chloride concentration (Table 1). Concerns that large amounts of Ringer's lactate infusion can increase plasma lactate levels in critically ill patients have led to the development of Ringer's acetate, in which the lactate buffer is replaced by acetate.^(30)^ Thus, the composition of Ringer's lactate and Ringer's acetate is almost identical with the exception of the added buffer (lactate or acetate, respectively) (Table 1).

Plasma-Lyte is another balanced solution with an osmolality of 294mOsm/L and sodium and chloride concentrations of 140mmol/L and of 98mmol/L, respectively. Other electrolytes and buffers present in this solution include potassium, magnesium, acetate and gluconate (Table 1). In the next sections, the current evidence comparing balanced and unbalanced crystalloids in experimental models (Table 2) as well as in clinical studies involving healthy volunteers and septic and non-septic critically ill patients (Table 3) is presented.

## EXPERIMENTAL STUDIES

Most experimental studies comparing a balanced solution, in general Ringer's lactate or Plasma-Lyte, to an unbalanced solution (0.9% saline) were performed in animal models of hemorrhagic shock^(15-22)^ (Table 2). While resuscitation with 0.9% saline, but not with a balanced solution, led to hyperchloremic metabolic acidosis,^(15-22)^ renal blood flow and kidney oxygen consumption were improved with Plasma-Lyte resuscitation^(20)^ (Table 2).

Plasma-Lyte was compared to 0.9% saline only in one experimental model of abdominal sepsis (Table 2).^(14)^ In this study, rats were randomly allocated to resuscitation with either Plasma-Lyte or 0.9% saline, subcutaneously, eighteen hours after a cecal ligation and puncture.^(14)^ Resuscitation with Plasma-Lyte was associated with maintained plasma chloride levels and arterial pH, lower plasma creatinine, lower urinary cystatin C, lower neutrophil gelatinase-associated lipocalin (NGAL), lower plasma interleukin-6 (IL-6), lower incidence (and severity) of acute kidney injury and a higher survival rate than animals resuscitated with 0.9% saline. Serum potassium levels, which are a major concern related to balanced crystalloids containing potassium, did not differ between the groups.^(14)^

## STUDIES INVOLVING HEALTHY VOLUNTEERS

Four randomized crossover studies addressed the effects of 0.9% saline, Plasma-Lyte, Ringer's lactate or Hartmann's solution on acid-base balance and electrolyte disorders in healthy volunteers.^(44-47)^ All studies reported hyperchloremic metabolic acidosis following a 0.9% saline infusion.^(44-47)^

While 50mL/kg of Ringer's lactate infusion transiently decreased serum osmolality and increased venous pH in healthy volunteers, a lower urinary output was seen after the same amount of 0.9% saline infusion.^(44)^ In another study, Reid et al. infused two liters of 0.9% saline or Hartmann's solution for two hours in healthy volunteers on two separate occasions.^(45)^ In addition to a more pronounced and sustained intravascular volume expansion with 0.9% saline than with Hartmann's solution, urinary output was lower with 0.9% saline than with Hartmann's solution.^(45)^ The same group compared 0.9% saline with Plasma-Lyte (two liters within one hour) in twelve healthy volunteers on two separate occasions (up to 10 days apart).^(46)^ In this study, Plasma-Lyte and 0.9% saline produced similar intravascular volume expansion. Nevertheless, 0.9% saline yielded sustained hyperchloremia, reduced SID, increased extravascular volume (edema) and lowered diuresis compared with Plasma-Lyte.^(46)^ Additionally, renal artery flow velocity and renal cortical perfusion assessed with magnetic resonance imaging were significantly lower after 0.9% saline administration than after Plasma-Lyte. There was no difference in urinary NGAL.^(46)^

## STUDIES IN SEPTIC AND NON-SEPTIC CRITICALLY ILL PATIENTS

There is limited data available supporting the use of balanced solutions in septic shock patients.^(7,8,10)^ A meta-analysis including fourteen studies with 18,916 adult septic patients suggested that resuscitation with balanced crystalloids compared with unbalanced crystalloids (0.9% saline) may be associated with a lower mortality rate (odds ratio - OR, 0.78; 95% confidence interval - 95%CI, 0.58 to 1.05).^(7)^ More recently, another network meta-analysis that included ten randomized clinical trials with 6,664 septic patients showed no significant difference in the need for renal replacement therapy (RRT) between balanced crystalloids and 0.9% saline (OR, 0.85; 95% CI, 0.56 to 1.30).^(8)^

Most studies comparing balanced and unbalanced crystalloids involved a mixed sample of clinical-surgical critically ill patients^(23-37,48-51)^ (Table 3). The safety and efficacy of volume expansion with a balanced crystalloid (Plasma-Lyte 148) compared with that of 0.9% saline were evaluated in a prospective, exploratory, cluster-randomized, blinded, double-crossover trial.^(49)^ In this study, involving 2,278 critically ill patients, a median volume infusion of 2 liters of balanced crystalloid or 0.9% saline did not affect the risk of acute kidney injury (AKI) according to RIFLE (Risk, Injury, Failure, Loss, and End-Stage) classification (relative risk - RR, 1.04; 95%CI, 0.80 to 1.36; p = 0.77), the need for RRT (RR, 0.96; 95%CI, 0.62 to 1.50; p = 0.91), ICU (RR, 0.92; 95%CI, 0.68 to 1.24; p = 0.62) or in-hospital mortality (RR, 0.88; 95%CI, 0.67 to 1.17; p = 0.40).^(49)^ Nevertheless, very few septic patients were included in this study, and acid-base and electrolyte parameters were not provided. This precluded determination regarding how much physiologic differentiation might have in fact occurred between the groups.^(49)^ Furthermore, the effects on the primary outcome and other secondary binary outcomes were assessed with simple chi-squared tests, ignoring the lack of independence of each patient's observation caused by the cluster-randomized design of the study.^(52)^ Consequently, the p-values were artificially high, and the 95% CI was over-narrowed.

A chloride-liberal strategy was compared with a chloride-restrictive strategy among critically ill adult patients in a before-after study.^(50)^ During a six-month control period (chloride-liberal period), 760 patients received intravenous fluids (0.9% saline, 4% succinylated gelatin solution or 4% albumin) according to the clinician's preference. After a 6-month interval, 773 patients received only chloride poor fluids (Hartmann's solution, Plasma-Lyte or 20% albumin).^(50)^ The authors demonstrated a significant decrease in acute kidney injury and failure (from 14.0% to 8.4%; p < 0.001) according to RIFLE classification and the need for RRT (from 10.0% to 6.3%; p = 0.005). No differences in in-hospital mortality or other clinical outcomes were observed.^(50)^ Contradictory findings were presented in a retrospective cohort study including 53,448 septic patients.^(48)^ In this observational study, resuscitation with balanced crystalloids, but not with unbalanced crystalloids, was associated with decreased risk of in-hospital mortality (RR, 0.86; 95%CI, 0.78 to 0.94; p = 0.001). Nevertheless, no significant difference in the incidence of acute kidney injury, need for RRT, and hospital and ICU lengths of stay was reported.^(48)^

A propensity-matched cohort study with 3,116 hospitalized patients with a systemic inflammatory response syndrome (SIRS) showed that balance crystalloids (Plasma-Lyte or Normosol), compared with 0.9% saline, were associated with a lower rate of major complications (atrial fibrillation, congestive heart failure, acute respiratory failure, pneumonia, sepsis and coagulopathy), a lower frequency of electrolyte abnormalities and hyperchloremic acidosis, shorter length of hospital stay, less need for hospital re-admission, and lower in-hospital mortality.^(51)^ Nevertheless, the incidence of acute kidney injury did not differ between the groups studied.^(51)^

Several small randomized trials compared balanced crystalloids with 0.9% saline^(23-37)^ (Table 3). In most trials, 0.9% saline induced hyperchloremic metabolic acidosis compared with either Ringer's lactate or Plasma-Lyte (Table 3). The effect of 0.9% saline versus Plasma-Lyte on coagulation (thromboelastography) was recently evaluated in eighteen trauma patients.^(27)^ The time from 2 to 20mm amplitude (K) was shorter, and the α angle higher after intravascular expansion with Plasma-Lyte than with 0.9% saline.^(27)^ Coagulation derangements secondary to crystalloid infusion may have clinical implications, as suggested by another study involving 66 patients undergoing aortic reconstructive surgery.^(24)^ In this study, patients who received 0.9% saline needed more platelets and blood product transfusion than did those who received Ringer's lactate.^(24)^ The effect of intravascular volume expansion with low-chloride versus high-chloride content crystalloids in critically ill or surgical patients was recently addressed in a meta-analysis.^(53)^ Twenty-one studies (15 randomized controlled trials) with 6,253 patients were included. Although high-chloride containing crystalloids did not affect mortality, they increased the risk of hyperchloremia and metabolic acidosis (risk ratio, 2.87; 95%CI, 1.95 to 4.21; p < 0.001) and the risk of acute kidney injury (risk ratio, 1.64; 95%CI, 1.27 to 2.13; p < 0.001).^(53)^ Finally, there was an increase in blood transfusion volume following resuscitation with 0.9% saline compared with low-chloride crystalloids.^(53)^

In summary, the current literature suggests that resuscitation of septic and non-septic critically ill patients with unbalanced crystalloids, mainly 0.9% saline, is associated with a higher incidence of acid-base balance and electrolyte derangements. Most importantly, resuscitation with unbalanced crystalloids might be associated with increased bleeding risk, an increased need for transfusion, a higher incidence of acute kidney injury, an increased need for RRT and increased mortality.

## FUTURE DIRECTIONS

Although it appears that all crystalloid solutions have similar hemodynamic effects,^(10)^ the impact of intravascular volume expansion with balanced solutions on regional and microcirculatory blood flow, tissue perfusion, mitochondrial function, systemic inflammation and coagulation need to be further evaluated in both experimental and clinical studies.^(10)^ Furthermore, this review, as with any non-systematic narrative review, may have limitations in terms of the comprehensiveness of the search strategy used to identify relevant papers and the lack of standardization of methods for data extraction, analysis and interpretation. Thus, systematic reviews with meta-analysis on the subject are warranted.

## CONCLUSION

Adequate fluid replacement is crucial to maintain perfusion pressure and, ultimately, tissue perfusion in septic shock patients. Although the current sepsis guidelines recommend crystalloids as first-line fluids for septic shock resuscitation, in the light of the inconclusive nature of the available literature, no definitive recommendations on the most appropriate crystalloid solution can be made. Therefore, the safety and efficacy of balanced solutions, compared with 0.9% saline, for septic shock resuscitation should be further evaluated in a large, multicenter, pragmatic, randomized clinical trial.
